# The morphological and ecological variation of *Arctostaphylos* (Ericaceae) fruit: A link between plant ecology and animal foraging behavior

**DOI:** 10.1002/ece3.9801

**Published:** 2023-03-15

**Authors:** Rebecca E. Crowe, V. Thomas Parker

**Affiliations:** ^1^ San Francisco State University San Francisco California USA; ^2^ UCI Herbarium University of California, Irvine Irvine California USA

**Keywords:** dispersal, fire regime, fruit morphology, generalized mixed‐effects model, scatter‐hoarding rodents, seed viability

## Abstract

Persistent soil seed banks are characteristic of *Arctostaphylos* (Ericaceae) species in the Mediterranean‐climate California Floristic Province. While most species are obligate seeders, regeneration of stands of all *Arctostaphylos* species ultimately depends on post‐fire seedling recruitment. *Arctostaphylos* seed banks are created, in large part, by scatter‐hoarding rodents. Variation in fruit morphology, therefore, is expected to impact the *Arctostaphylos*–rodent interaction. Seeds produce sufficient rewards (nutritious mature embryo) to entice rodents to disperse and ultimately bury seeds in the soil. Hard seed coats increase the time required to extract the embryo, encouraging rodents to choose storage over immediate predation, and nutlets are frequently empty. We assessed the variation of fruit nutlet fusion and seed viability among 38 *Arctostaphylos* taxa. Factors such as latitude, elevation, life history, ploidy, and phylogenetic position were also analyzed. Generalized mixed‐effects models were used to determine the factors contributing to variation in fruit nutlet fusion and seed viability. Our results indicate that fruit volume and shape are the most important variables affecting nutlet fusion and seed viability. Additionally, other potential influences only show a weak correlation and are not predicted to significantly impact nutlet fusion or seed viability. These findings provide insights into evolved strategies used by plants to increase reproductive success via scatter‐hoarding rodents. Our study benefits the conservation and restoration of *Arctostaphylos* stands by emphasizing the importance of animal‐mediated dispersal and providing estimates of seed viability for different species. With the anticipated effects of climate change, such as departures from historic fire regimes, the preservation of the relationship between plants and animal foragers is crucial for the continued survival of *Arctostaphylos* and California's evergreen chaparral.

## Graphical abstract

We assessed the variation of fruit nutlet fusion and seed viability among 38 Arctostaphylos taxa. Our results indicate that fruit volume and shape are the most important variables affecting nutlet fusion and seed viability. These findings provide insight into evolved strategies used by plants to increase reproductive success via scatter‐hoarding rodents.

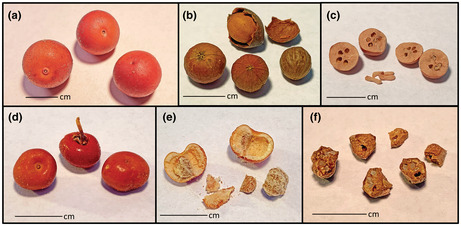

## 1 INTRODUCTION AND BACKGROUND

The reciprocal interactions between scatter‐hoarding rodents and plants create a challenge for plants with long‐term persistent seed banks. The interactions between plants and their dispersal vectors influence fruit and seed morphology and chemistry, regardless of the type of vector (Rubio de Casas et al., 2015; Tiffney, 2004), and determine propagule dispersal, a significant part of a plant's life history and critical to the dynamics of plant communities (Levine & Murrell, 2003; Venable & Brown, 1988; Wang & Smith, 2002). Scatter hoarding represents a particular type of dispersal in which animals collect, bury, and manage propagules in numerous small caches in the soil over long time periods (Vander Wall, 2001) and fruit and seed trait patterns provide important insights about selection by rodents and how those traits in turn may be influencing or reinforcing animal behavior (Vander Wall, 2010). But scatter‐hoarding rodents are both consumers and dispersers of seed and fruit, and thus variation in propagule traits influences the balance between those processes (Wang et al., 2012). We focus here on morphological variation in fruit and seed among species in the plant genus *Arctostaphylos* (Ericaceae) whose fruit and seed are buried by scatter‐hoarding rodents (Moore & Vander Wall, 2015; Parker, 2015).

A key issue for *Arctostaphylos* species in the Mediterranean‐climate California Floristic Province is that their seed is physiologically dormant until fire‐stimulated (Keeley, 1977; Parker & Kelly, 1989). Animals select seeds for caching based on traits including size, nutrient composition, secondary metabolites, perishability, handling costs, and infestation by insects (Lichti et al., 2017). Studies indicate some variability among rodent species, but in general, size and perishability are principally considered when there are conflicts among favored traits (Chang et al., 2009; Hadj‐Chikh et al., 1996; Reichman, 1988). Whether scatter‐hoarding animals directly assess seed viability is unknown, but seeds that are cached by rodents are predominantly viable (Jansen et al., 2004) and some rodents notice and reject insect‐infested seeds preferring intact and viable seeds (Perea et al., 2012). Smaller seeds are more likely to experience predation soon after they are discovered, while larger seeds are more often collected and dispersed (Jansen et al., 2002; Muñoz & Bonal, 2008a; Vander Wall, 2010; Wang et al., 2012).

How scatter‐hoarding rodents and dormant plant propagules interact is important for understanding seed dispersal and survival. In grasslands, plants with persistent seed banks sustain less predation than those with transient seed banks (Hulme, 1998). In forest systems, rodents appear to favor caching seed with short‐term dormancy (<1 year) when selecting between dormant versus rapidly germinable seed (Chauvet et al., 2004; Fox, 1982; Jansen et al., 2006). In the case of mesic temperate zone oaks interacting with squirrels and other rodents, fall germinating white oaks are generally consumed immediately, while acorns of red oaks that delay germination a few months to early spring are cached (Chang et al., 2009; Fox, 1982; Hadj‐Chikh et al., 1996; Lichti et al., 2017; Steele et al., 2001; Xiao et al., 2009). In some tropical trees, propagules with longer dormancy are cached (Chauvet et al., 2004) or the embryo is excised prior to caching if the seed readily germinates (Jansen et al., 2006).

The time frame for long‐term seed dormancy, however, is considerably different from that for transient or short‐term dormancy as in red oaks or other species. In the case of fire response species like *Arctostaphylos*, germination only occurs post‐fire and results in time intervals of decades between germination events (Keeley & Fotheringham, 2000; Parker & Stickrod, 2022; Pausas & Lamont, 2022). One issue is whether there might be traits that have responded to rodent predation to influence the balance between consumption and caching in *Arctostaphylos*. Traits that have been found to favor caching, like dormancy and size, should have a net influence on long‐term seed bank densities. Yet, in *Arctostaphylos,* seed bank density is negatively related to fruit and seed size (Parker & Ingalls, 2022).

**FIGURE 1:**
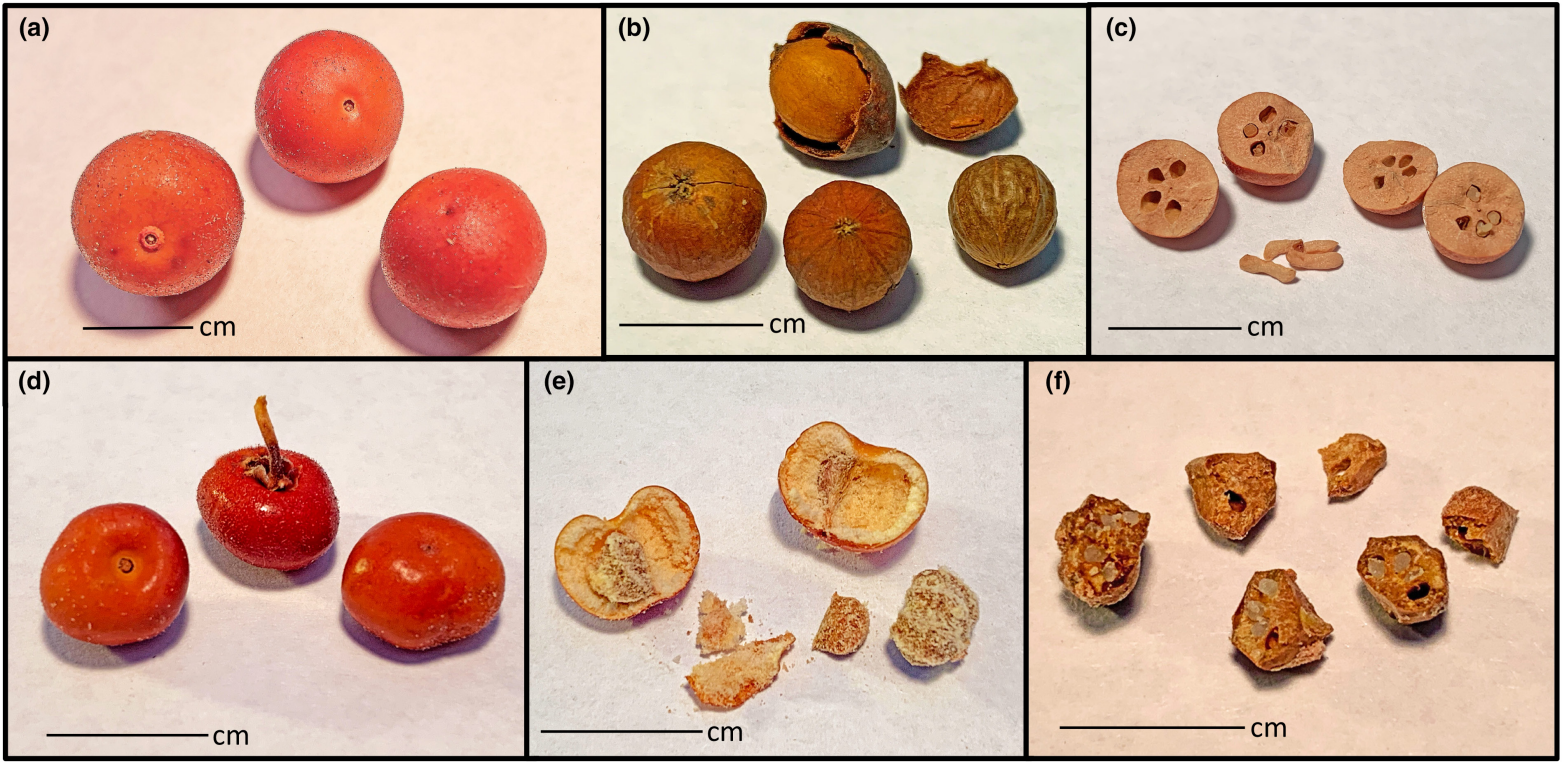
Fruit and nutlets of two *Arctostaphylos* species. (a) *A. glauca* fruit; (b) *A. glauca* nutlets with one showing the exocarp; (c) two fused sets of *A. glauca* nutlets opened showing filled and empty chambers with a few seed below; (d) *A. glandulosa* fruit; (e) one *A. glandulosa* fruit opened to show partially fused nutlets and mealy mesocarp; (f) a few dissected *A. glandulosa* nutlets, some fused together, seed chambers either empty or with seed; seed appears as the whitish solid in the chambers.

Rodents tend to disperse whole *Arctostaphylos* fruit (Parker et al., 2015; Parker & Ingalls, 2022) but they also will cache nutlets separated from fruit*,* including nutlets found in coyote or bear scat (Moore & Vander Wall, 2015). While initially size appears to be the only trait that varies among species in *Arctostaphylos*, several dimensions of variability among fruit and seed might influence scatter‐hoarding rodents. First, the morphology of *Arctostaphylos* fruit varies in shape as well as in size, with *Arctostaphylos* producing dry, multi‐seeded drupes that range between spheric and vertically depressed‐spheric in shape (Figure 1). The drupes also range in size from around 3–4 mm in some species and up to 10–16 mm wide in others (Parker et al., 2012). The mature fruit are composed of three layers. The outer layer, the exocarp, is dry and thin and ranges in color, glands, and pubescence. The mesocarp is also dry, and the composition and thickness vary among species from thick and mealy to absent. The third layer is composed of seeds surrounded individually by thickened, stony endocarps. The collective unit of the hardened endocarp and an individual seed resemble small nuts and are called nutlets. The nutlets are wedge‐shaped and are either fused together along radial surfaces into a single spherical unit, or weakly fused such that nutlets may variably separate from each other after dispersal. Nutlet traits thus create a second level of diversity for rodents as the variable fusion of adjacent nutlets create different sizes of potential rewards. Finally, not all ovules develop into mature seeds, creating a third level of variability for rodent assessment, and mature fruit usually contains a mix of empty and seed‐filled nutlets.

Our objective in this study is to examine variation in fruit and nutlet characters for patterns that may influence rodent decision‐making; specifically, to assess and document the variability in size, nutlet fusion, and seed viability in these taxa, as a first step to understanding the development of these significant seed bank densities. Relationships between traits and seed bank densities might reveal traits responding to rodent predation to influence the balance between consumption and caching in *Arctostaphylos*. Here, we examined fruit: (1) for differences in nutlet fusion and seed viability among species; (2) to determine the extent to which fusion of nutlets and seed viability are influenced by morphology, environmental factors, life history characteristics, and species identity.

## 2 MATERIALS AND METHODS

### 2.1 Data collection

*Arctostaphylos* fruit mature in early to mid‐summer at lower elevations, delaying to late summer to early fall at higher elevations; consequently, fruits were collected from chaparral and mixed evergreen forests throughout California during the summers and falls of 2013–2016. Fruits are at their peak size and dry at maturity. Collections from previous studies collected from Northern and Central California sites during 1988–2013 were also opportunistically used in this study. In most of the newer collections, fruits were separated by mother plant and population, but in older collections, fruits had been consolidated. Older collections used a consistent protocol: many fruits were collected from many individual plants (usually 10–20 plants) from one population and pooled. Fruits used from the older study allowed us to include species that are difficult to obtain because of rarity or accessibility. Once collected, all fruits were stored at room temperature. We aimed to include species with different fruit sizes, life histories, phylogenetic positions (clade), and ploidy so that as many different species as feasible were sampled per combination of factors (Table 1).

**TABLE 1:** Species sampled by clade, ploidy level, and life history.

Clade	Ploidy	Life history	*Arctostaphylos* taxon
Large	2 n	Resprouter	***A. rainbowensis***
		Non‐sprouter	*A. andersonii, **A. canescens**, A. columbiana, A. gabilanensis, **A. glauca**, A. glutinosa, A. klamathensis, A. montaraensis, A. nortensis, A. obispoensis, A. pajaroensis, **A. pechoensis**, A. pumila, A. pungens, A. purissima* subsp. *globosa, A. purissima* subsp. *purissima, **A. refugioensis**, A. regismontana, A. virgata, A. viscida* subsp. *pulchella, A. viscida* subsp. *viscida*
	4 n	Resprouter	***A. crustacea subsp.* *crustacea*** *, A. glandulosa* subsp. *cushingiana,* ***A. glandulosa subsp.* *leucophylla*** *, A. glandulosa* subsp. *glandulosa, A. manzanita* subsp. *roofii,* ***A. nevadensis* subsp.** ***knightii*** *, tomentosa*
		Non‐sprouter	***A. manzanita* subsp. *elegans*** *, A. manzanita* subsp. *manzanita,* ***A. montana* subsp. *montana*** *,* ***A. nevadensis* subsp. *nevadensis***
Small	2 n	Resprouter	***A. patula, A. rudis***
		Non‐sprouter	***A. hookeri**, A. ohloneana, **A. stanfordiana***
	4 n	Resprouter	none sampled
		Non‐sprouter	no known taxa exist with this combination of traits

*Note*: All taxa were sampled for height, width, volume, and nutlet fusion. The bold‐faced taxa were also sampled for seed viability.

**FIGURE A1:**
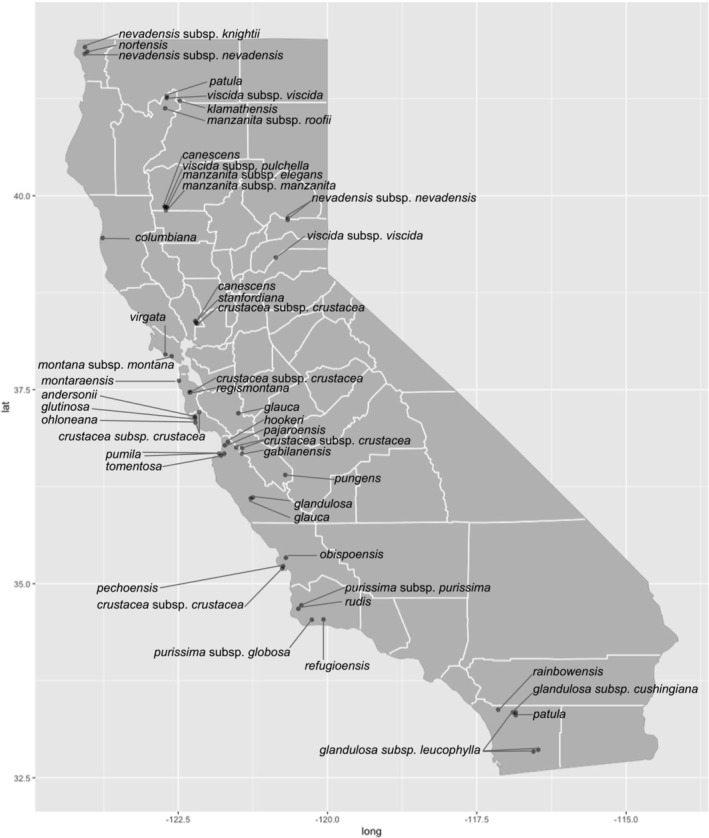
Map of sampling locations.

Fruit and, when feasible, vouchers were collected from 38 *Arctostaphylos* taxa (Figure A1). Voucher specimens were deposited at the H.D. Thiers Herbarium (SFSU) with duplicates at the U.C. Irvine Herbarium (IRVC). Herbarium data are available through the Consortium of California Herbarium (Consortium of California Herbaria (CCH), 2018). Specimens were identified using the *Flora of North America*, *The Jepson Manual*, and *Field Guide to Manzanitas (*Kauffmann et al., 2015 *;* Parker et al., 2009, 2012 *)*. A minimum of 33 mature, dry, undamaged fruits were sampled from each population, and from multiple individual plants when possible (Table A1). For example, for *A. pechoensis*, three fruits from 11 individual plants from one population were sampled and measured, totaling 33 fruits measured. In a handful of species, we were only able to sample from 2–3 individuals due to year‐to‐year variation in the onset of fruiting and duration of fruiting. For example, in a remote population of *A. nevadensis* subsp. *knightii*, we scoured the population at the time we expected the population to be in fruit, and found just two plants with fruit. Because the site was remote, we were not able to make reconnaissance trips to assess the fruiting time and missed peak fruiting time. However, most species had 8–10 individual plants sampled. Fruit was brought into the lab at San Francisco State University for measurement of fruit morphologic characters.

We counted the number of fused nutlets and viable seeds to quantify the extent of variation between species and to identify potential drivers of this variation. For each fruit, we counted the total number of nutlets and the number of fused nutlets. We examined each nutlet or conglomerate of fused nutlets to determine the number of apparent seed chambers, which are the ovary locules (Figure 1). Seed chambers were interpreted as discreet segments delineated either by complete separation or by indentations on surface caused by the fusion of nutlets. The number of seed chambers were confirmed by radially cutting fused nutlet segments in half with a jeweler's hacksaw. We counted the total number of nutlets and number of nutlets with at least one radial surface fused for each fruit and used this count to derive % nutlet fusion (# fused chambers/ # total chambers) for each fruit. The proportion of viable seeds per fruit was measured as the number of full and plump seeds (=viable seeds) to the # of total seed chambers in one fruit. We measured % seed viability for a subset of 15 *Arctostaphylos* taxa. Ten of those species had fruit that separately was collected from multiple individuals, while the remaining five species were from the older collections. Measurements of seed viability were made on the same fruit we measured to calculate the % nutlet fusion.

Effective propagule size will vary not only with fruit size but also post burial based on the degree of nutlet fusion, because fruits with some free nutlets will functionally become multiple smaller propagules as the exocarp and mesocarp are removed due to decay or by animals. Processes that influence fruit size may in turn influence nutlet fusion in *Arctostaphylos*. Previous fruit size studies explored the drivers of fruit size differences across local to global spatial scales. These studies indicated that elevation, latitude, and “droughtiness” influence seed size (Baker, 1972; Moles et al., 2007; Simpson et al., 2017). Potential environmental drivers of variability in nutlet fusion include elevation, average yearly precipitation, average yearly maximum temperature, climate water deficit, latitude, and longitude. These data were extracted for each population from the PRISM 30 years normal baseline dataset that describes the average monthly and annual conditions from 1981 to 2010, and the Climatic Water Deficit dataset, which is an estimate of drought stress on soils and plants (Table A2) (PRISM Climate Group, n.d.; Flint & Flint, 2014). Climate data were extracted from the PRISM dataset using a single GPS point for each population.

In addition to environmental variables, species and fruit‐level traits were also measured. The life history and ploidy level of species may potentially influence nutlet fusion and seed viability. Differences in seed viability and percent seed set between resprouters and non‐sprouters and ploidy level have been documented (Keeley & Keeley, 1977; Kelly & Parker, 1991). Life history, phylogenetic position (based on Wahlert et al., 2009), and ploidy level of each taxon were recorded as categorical variables. Each categorical variable had two levels: non‐sprouting or resprouting, large or small clade, and diploid or tetraploid.

We also measured six morphological traits of potential ecological importance. We made morphological measurements on the same fruit used to calculate % nutlet fusion and % seed viability. Fruit height and width were measured, in millimeters, with digital calipers (Mitutoyo Digimatic CD‐6”BS); height was measured at the tallest point of the fruit, between the peduncle and the flower end, and width was measured at the widest place along the fruit equator. Fruit shape was calculated as the ratio of fruit height to width and corresponds to the following conventional 3‐d shapes: obloid/ellipsoid (2:1), widely ellipsoid (6:5), globose (1), depressed globose (3:4), oblate (1:2) (Simpson, 2010). Fruit volume was calculated as the volume of an oblate spheroid (Simpson, 2009). Pulp, the mealy mesocarp layer of fruit present in some *Arctostaphylos* species, was assessed and assigned a category: thick sheet, scant, absent, powdery, mealy/powdery, mealy, and appressed sheet. Morphological fruit traits are summarized by species in (Table A3).

### 2.2 Data analysis

**FIGURE A2:**
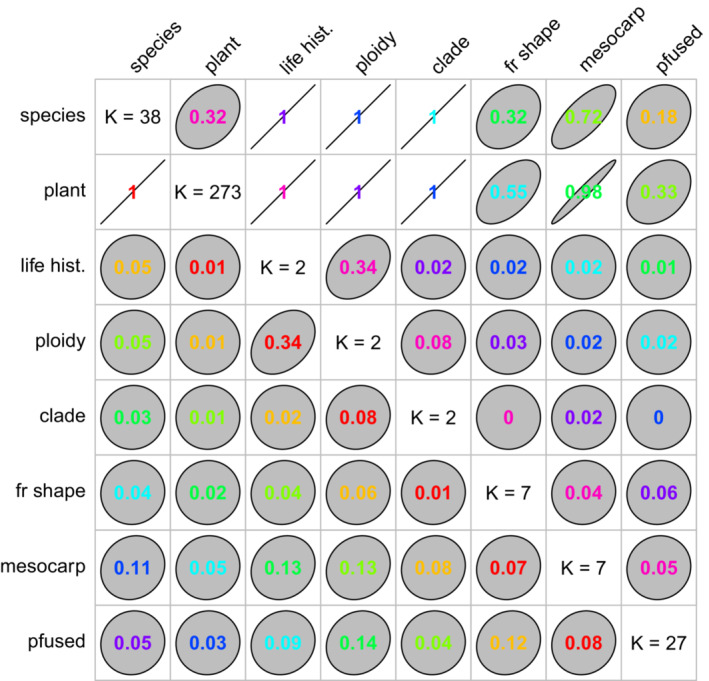
Goodman and Kruskal's tau measure for nutlet fusion. We used Goodman Kruskal's tau measure for associations between categorical variables and endocarp fusion. The greatest association was found with fusion and plant, which is the individual plant that fruit was collected from. Species and plant are correlated with each other and life history, ploidy, and clade. Plant is also correlated with mesocarp. Variables with strong associations should not be used together as fixed effects. All other variables are not correlated with one another.

**FIGURE A3:**
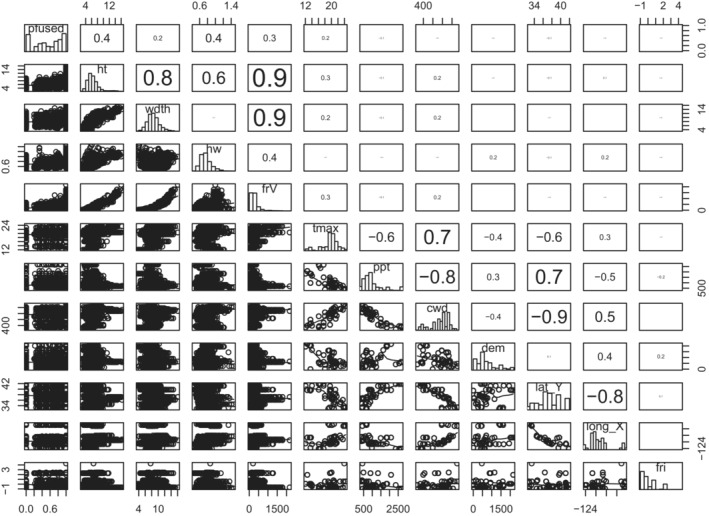
Pearson correlations for nutlet fusion We used Pearson correlations to check for potential correlations of fruit fusion and continuous variables of interest and between explanatory variables, presented as a matrix. Variables from left to right (and repeat from top to bottom) are: % fused endocarp, height, width, ratio of height to width, fruit volume, prior 20 years average maximum temperature, precipitation, climatic water deficit, elevation, latitude, longitude, fire return interval. There are no strong correlations between fruit fusion (pfused) and the explanatory variables. Large numbers indicate strong correlations and small or absent numbers indicate very weak correlations. There are weak correlations with height (ht) (0.4), height to width (h:w) (0.4), and tmax (0.3). There are strong correlations (Pearsons, 0.8 or higher) between some environmental variables; height with width, frV; width with frV; ppt with cwd; cwd with lat; lat with long. This indicates that covariables are collinear and should not be included together in a model. Pearson correlations are moderately high (0.7) for tmax with cwd and ppt with lat.

**FIGURE A4:**
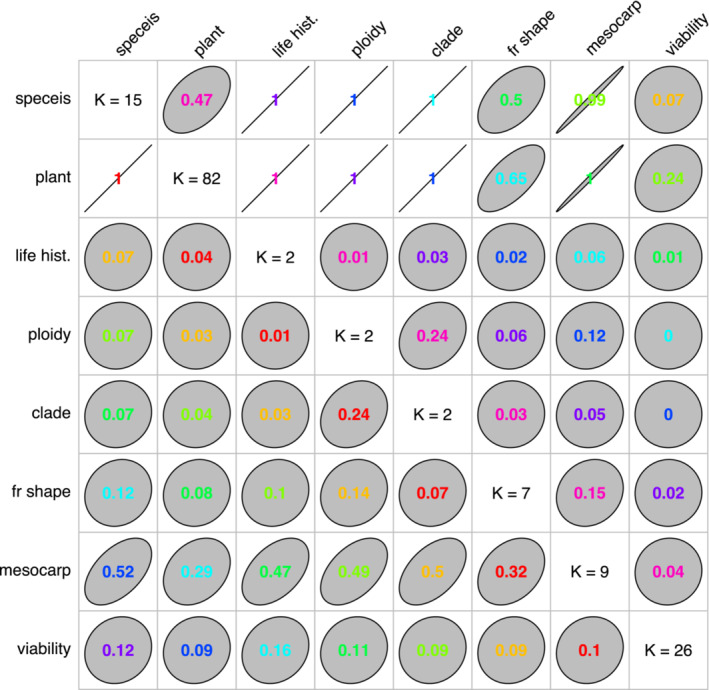
Goodman and Kruskal's tau measure for seed viability. We used Goodman Kruskal's tau measure for associations between categorical variables and seed viability. The greatest association was found with viability and plant, but the strength of the association is weak. Species and life history, ploidy, clade, and mesocarp are strongly associated. Plant is strongly associated with species, life history, ploidy, clade, and mesocarp. All other variables are not correlated with one another.

**FIGURE A5:**
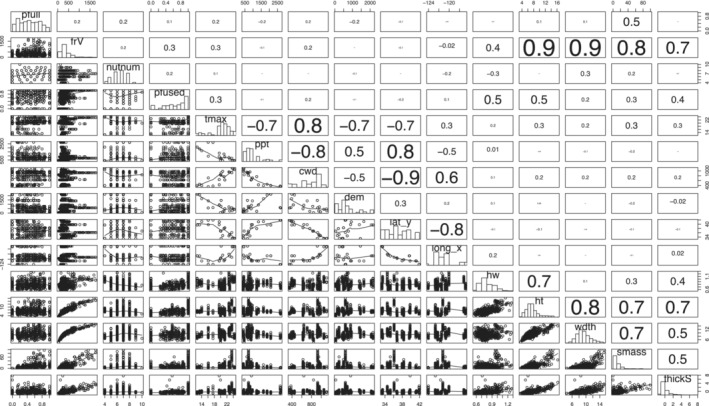
Pearson correlations for seed viability. We used Pearson correlations to check for potential correlations of seed viability and continuous variables of interest and between explanatory variables, presented as a matrix. Variables from left to right (and repeat from top to bottom) are: % viable seed, fruit volume, nutlet number, % fused endocarp, prior 20 years average maximum temperature, precipitation, climatic water deficit, elevation, latitude, longitude, ratio of height to width, height, width, seed mass, and endocarp thickness. There are no strong correlations between seed viability and the explanatory variables. Large numbers indicate strong correlations and small or absent numbers indicate very weak correlations.

All analyses were conducted using the R statistical software (R Core Team, 2018). We analyzed the nutlet fusion and seed viability dataset separately, and first we describe the nutlet fusion data analysis methods. We explored the data with plots to detect outliers, homogeneous variance of independent variables, normality of independent variables, collinearity among covariates (Figures A2, A3, A4, A5), too many zeros, and independent y variables (Zuur et al., 2010). Data exploration confirmed a non‐normal distribution of data, non‐independent observations, and heterogeneous variance indicating the need to use GLMMs (generalized linear mixed‐effects models). We used GLMMs to model relationships between fruit nutlet fusion, environmental, morphological, and life history parameters. The response variable was fruit nutlet fusion, which is proportional and includes zeros and ones, and best fit with a binomial distribution and logit‐link. Continuous independent variables were centered, mean subtracted from each observation, and scaled. Centering removes the correlation between slope and intercepts and makes the treatment main effects meaningful, that is, independent of the slopes (Schielzeth, 2010).

After “hard thinking” we created a set of candidate models based on our understanding of the drivers of effective propagule size (Burnham et al., 2011). We fit a global model for fruit nutlet fusion using the “lme4” package, determined the structure of the random effects, and lastly, determined the structure of the fixed effects (Bates et al., 2015; Burnham et al., 2011). We established the structure of the random‐effects part of the model using BIC (Bayesian Information Criterion) to rank several models with different combinations of random‐effect terms and the best model was: species nested in population with an observation‐level random effect (Ucode). Since we were not able to include multiple populations for all species, we decided to include population as a random effect to account for potential correlations but not interpret the variation of population‐level effects. Many zeros were detected in data exploration, so we included an observation‐level random effect (OLRE) to account for overdispersion. We checked for overdispersion and the OLRE seemed to adequately address overdispersion (chisq = 616, residual *df* = 1712).

To determine the structure for the fixed effects, we built and BIC ranked a set of candidate models. Model sets were developed with the question: do environmental, plant traits, or fruit traits explain variation in proportion nutlet fusion? Environmental models contained combinations of the following factors: elevation, precipitation, daily maximum temperature, climatic water deficit, latitude, longitude, and ecologically sensible two‐way interactions. Correlated factors were not used together in the same model; for example (for fusion), latitude and precipitation were highly correlated. Plant trait models included combinations of clade, ploidy, and life history. Fruit trait models included combinations of shape, fruit volumes, mesocarp, and ecologically sensible two‐way interactions. Maximal models included all plant and fruit trait factors along with combinations of uncorrelated environmental variables. The random effects of species identity, population, and unique identifier were held constant for all models. We compared the models using BIC and the best model has the lowest BIC.

Two models were within 7 BIC and the best model included fruit volume and fruit shape. The next best model was 6.11 BIC higher than the best model, which differed by including the interaction of fruit volume and shape. In other words, the model with fruit fusion and shape was 21 times more likely than the next model to be true (Table 2). While models up to 7 BIC different may be considered plausible (Burnham et al., 2011), we did not pursue model averaging because we were also interested in the variation between species (random effects), which is not accounted for in model averaging (Bolker et al., 2009).

**TABLE 2:** Model selection table for % nutlet fusion within fruitModels were ranked with BIC.

Model name	Intercept	Fruit volume	Fruit shape	Volume: Shape	Life history	Ploidy	Clade	DEM	CWD	Df	BIC	Delta	Weight
Fruit traits	0.81	0.52	0.55	−	−	−	−	−	−	6	5723.6	0.00	0.955
Fruit traits‐ interaction	0.79	0.53	0.58	0.12	−	−	−	−	−	7	5729.7	6.11	0.045
Maximal‐ CWD	0.91	0.52	0.55	−	+	+	+	−0.17	−0.31	11	5758.4	34.77	0

*Note*: Each row summarizes a single model and continuous variables included in the model have a numerical estimate (in log odds) and categorical variables included are indicated with “+”. The top two models and the best maximal model are presented. See supplemental table X for rankings of all models. The best model is: fruit fusion ~ volume (0.52) + shape (0.55).2313.

We utilized the same model fit and selection protocol for the viability dataset. However, we considered two additional variables in the fruit traits model sets: endocarp thickness and % nutlet fusion and did not use population as a random effect. We found that the best model included species as random effects and fruit volume, fruit shape, and the proportion of fused nutlet as fixed effects (Table 3). The next best model was 19.09 BIC higher than the best mode, and was comprised of fruit shape, proportion nutlet fusion, and endocarp thickness.

**TABLE 3:** Model selection table for % viability within fruit.

Model name	Intercept	Fruit volume	Fruit shape	% endocarp fusion	Endocarp thickness	Clade	Life history	Ploidy	DEM	Latitude	Df	BIC	Delta	Weight
Fruit traits‐ volume	−0.17	0.47	0.13	0.12	−	−	−	−	−	−	6	1684.2	0.00	1
Fruit traits‐ endocarp thickness	−0.16	−	0.16	0.17	−0.10	−	−	−	−	−	7	1703.3	19.09	0
Maximal	−0.36	0.43	0.13	0.12	−	+	+	+	−0.16	−0.27	11	1709.5	25.31	0

*Note*: Models were ranked with BIC. Each row summarizes a single model and continuous variables included in the model have a numerical estimate (in log odds) and categorical variables included are indicated with “+”. The top two models and the best maximal model are presented. See supplemental table X for rankings of all models. The model ranked as the best model is: fruit viability ~ volume (0.47) + shape (0.13) + % endocarp fusion (0.12).

We assessed the fit of the best models for fruit fusion and viability by checking for unexplained patterns in the mean and variance with plots of scaled residuals versus fitted values and input variables using the DHARMa package in R and other base R plots (Hartig, 2019).

## 3 RESULTS

### 3.1 Fruit morphology

**FIGURE 2:**
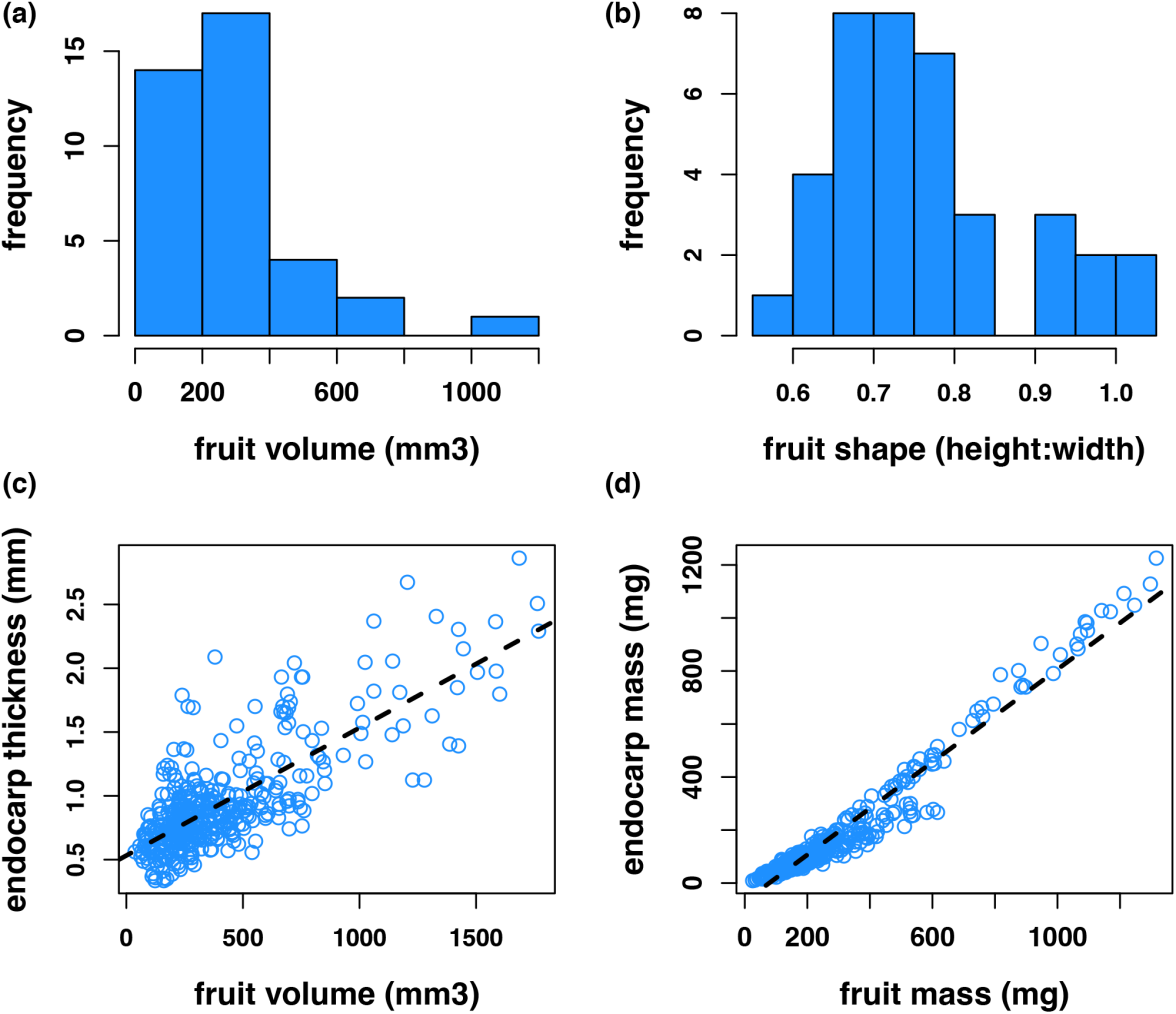
Descriptive aspects of *Arctostaphylos* fruit across species. (a) frequency in mean fruit volume for 40 taxa; (b) variation in mean fruit shape across 40 taxa; (c) relationship between endocarp thickness and fruit volume (adj R‐sq = 0.589, F_1,458_ = 658.3, *p* < 2.2 ^e‐16^); d) relationship between endocarp thickness and fruit mass (adj R‐sq = 0.95, F_1,458_ = 8715, *p* < 2.2 ^e‐16^).

**FIGURE 3:**
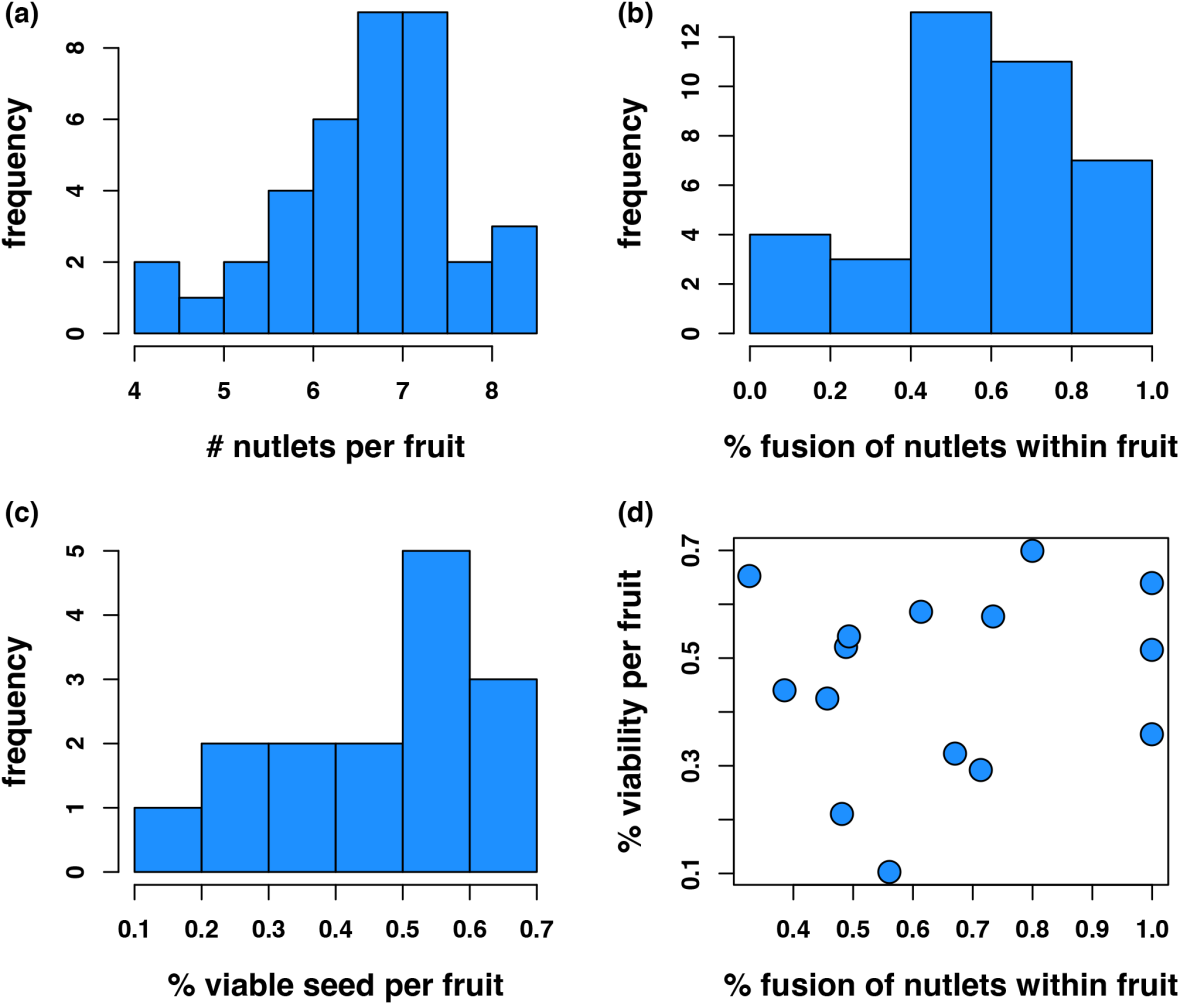
Variation in seed traits in *Arctostaphylos* fruit across species. (a) mean number of nutlets per fruit per species; (b) mean % fusion of nutlets per fruit per species; (c) mean % viable seed per fruit per species; (d) lack of relationship between % viability and % fusion of nutlets within fruit (adj R‐sq = 0.0718, F_1,13_ = 0.06219, *p* = .807).

We used the morphological data to examine the distribution of traits across species. For the taxa we examined, fruit volume was skewed toward smaller fruit (Figure 2a), while most species exhibited slightly flattened fruit (Figure 2b). As found in most studies, endocarp thickness and mass increased proportionately with the size and weight of the fruit (Figure 2c; adj R‐sq = 0.589, F_1,458_ = 658.3, *p* < 2.2 ^e‐16^) (2D; adj R‐sq = 0.95, F_1,458_ = 8715, *p* < 2.2 ^e‐16^). The mean percent fusion of nutlets within a fruit ranged across species from no or little fusion, to complete fusion (Figure 3a). For example, no nutlet fusion was observed in fruit of *A. nortensis*, and all nutlets were fused in fruit of *A. glauca, A. klamathensis, A. rainbowensis, and A. refugioensis* (Table A3). The mean number of nutlets formed within fruits ranged as well from a little over 4 to over 8 nutlets per fruit (Figure 3b).

**FIGURE 4:**
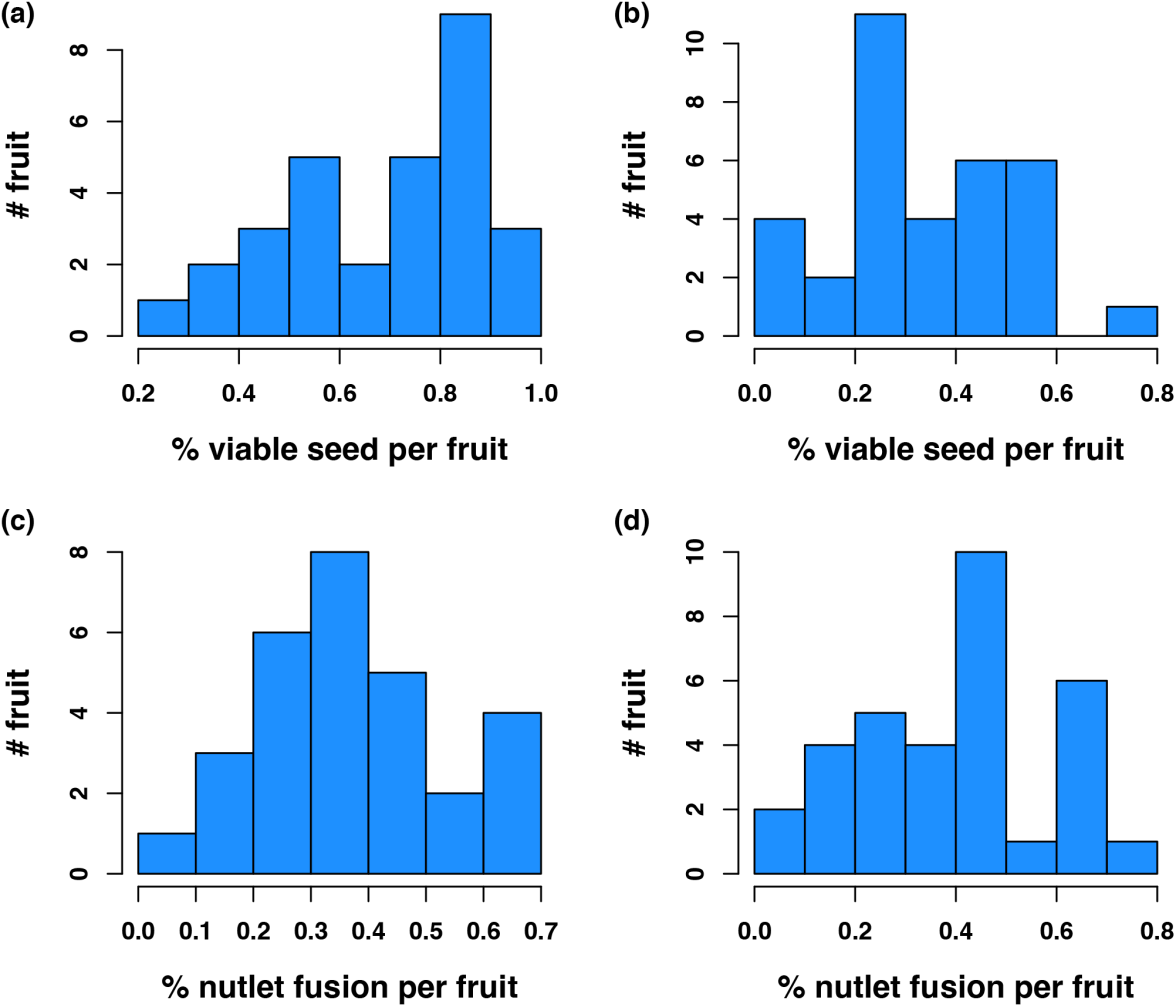
Within‐species variation in some fruit traits, examples from four taxa. (a) % viable seed per fruit for *A. stanfordiana*; (b) % viable seed per fruit for *A. rainbowensis*; (c) % nutlet fusion per fruit for *A. canescens*; (d) % nutlet fusion per fruit for *A. glandulosa* subsp. *leucophylla*.

Percent viability of these nutlets also ranged similarly (Figure 3c), but using species means, there was no relationship between percent fusion and percent seed viability among species (Figure 3d; adj R‐sq = 0.0718, F_1,13_ = 0.06219, *p* = .807). The standard deviation of these traits was fairly large for most species. The lowest amount of seed viability was found in *A. nevadensis* subsp. *knightii,* around 10.3% of the seeds in each fruit were viable, and the highest seed viability in *A. stanfordiana,* around 70% (Figure 3c, 4a,b, Table 4). Two examples of percent viability per fruit show considerable spread (Figure 4a,b) and these are representative of most taxa (Table 4). That type of variation in trait status was also true of percent nutlet fusion (except for those few species with complete fusion) and two examples are provided to show that variation (Figure 4c,d). Due to these ranges in trait values, we wondered if these were somewhat fixed within populations or varied; we tested for statistical differences in fruit traits among individuals of the same species within single populations and means among populations using one‐way ANOVAs. While the majority of *Arctostaphylos* species are local endemics comprising only one or a few populations, widespread species may experience considerably different climatic, edaphic, or animal influences and we selected 3 taxa for these analyses, *A. glandulosa* (# ind = 17, pop = 2), *A. viscida* (# ind = 21, pop = 2), and *A. crustacea* (# ind = 17, pop = 2). Statistically, these individuals and populations showed no difference in traits we were concerned with (% fusion, *A. glandulosa* individuals, *F* = 1.116, *p* = .367, populations, *F* = 1.39, *p* = .243; *A. crustacea* individuals, *F* = 1.672, *p* = .085, populations, *F* = 0.531, *p* = .469), although individuals of *A. viscida* were different (*A. viscida* individuals, *F* = 3.545, *p* = .0002, populations, *F* = 0.778, *p* = .381).

**TABLE 4:** Mean proportion viable seed per fruit by species, standard deviation in parentheses, number of fruits sampled per species.

Species	Mean proportion viable seed per fruit (SD)	Number of fruits sampled
*A. canescens*	0.652 (0.228)	30
*A. crustacea* subsp. *crustacea*	0.586 (0.276)	33
*A. glandulosa* subsp. *leucophylla*	0.323 (0.191)	33
*A. glauca*	0.639 (0.191)	29
*A. hookeri*	0.520 (0.280)	30
*A. manzanita* subsp. *elegans*	0.292 (0.245)	33
*A. montana* subsp. *montana*	0.577 (0.275)	30
*A. nevadensis* subsp. *knightii*	0.103 (0.135)	33
*A. nevadensis* subsp. *nevadensis*	0.418 (0.236)	33
*A. patula*	0.210 (0.223)	32
*A. pechoensis*	0.440 (0.308)	33
*A. rainbowensis*	0.355 (0.203)	34
*A. refugioensis*	0.515 (0.184)	33
*A. rudis*	0.540 (0.269)	33
*A. stanfordiana*	0.699 (0.204)	30

*Note*: Each species represents a single population.

### 3.2 Nutlet fusion within fruits model

**FIGURE 5:**
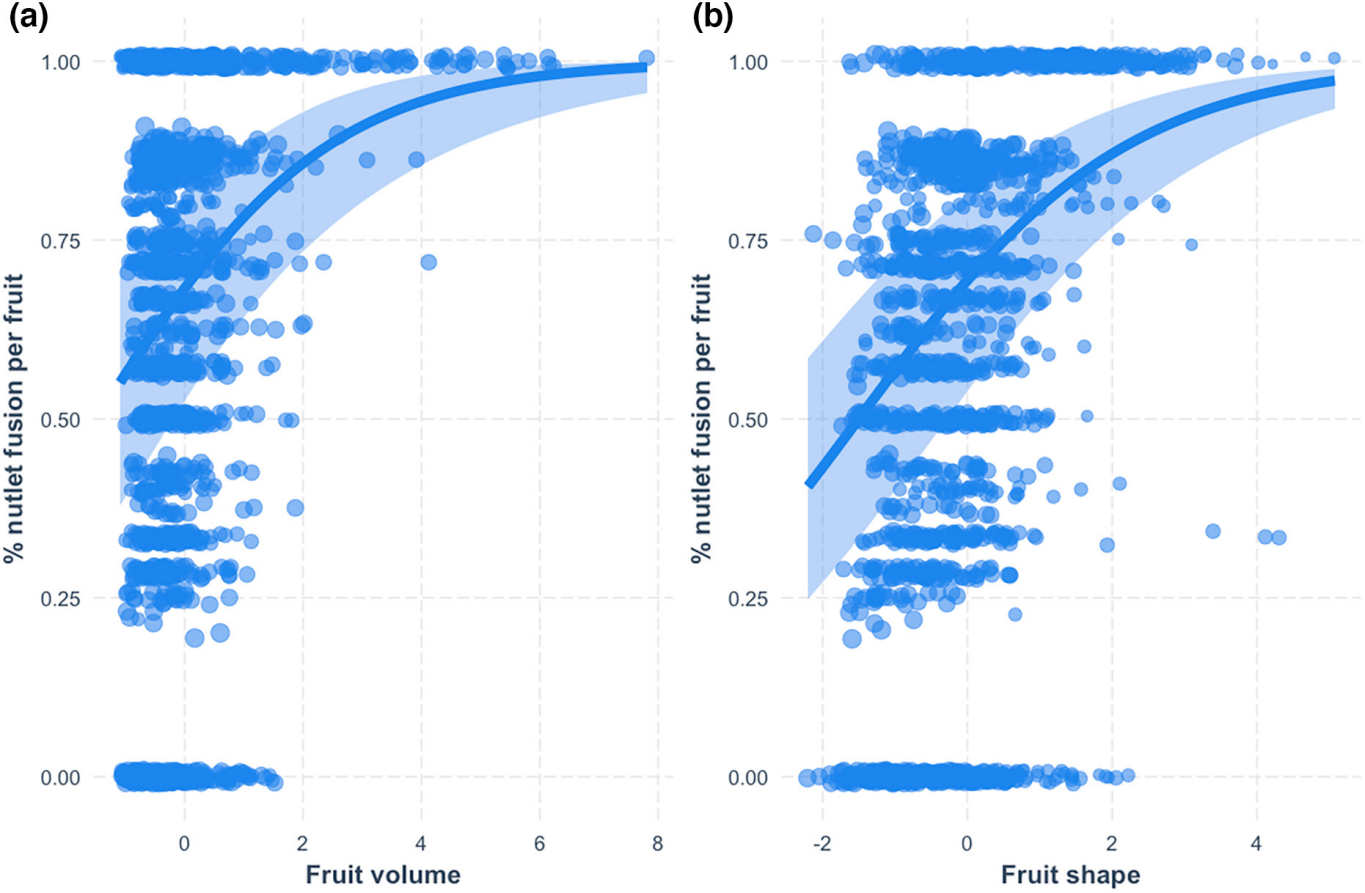
Regression estimates for the fruit nutlet fusion model, with observations plotted. The light blue ribbon corresponds to a 95% confidence interval. (a) Predicted probability of percent fused endocarp per fruit by fruit volume (in log‐odds). (b) Predicted probability of percent fused endocarp per fruit by fruit shape ratio (in log‐odds). Low values of height/width represent oblate‐shaped fruit, low‐medium values represent depressed globose fruit, medium values represent globose fruit, and high values represent obloid/ellipsoid fruit.

We learned from our nutlet fusion within fruits model that as fruit volume increased, the proportion of fused nutlets per fruit likewise increased. A one standard deviation (since we centered and scaled) increase in fruit volume was associated with a 0.514 unit increase in the expected log odds of percent fusion (se = 0.103, Profile CI: 0.328–0.715), or a predicted probability of 62.6%, when shape was held at an average value (Table 5, Figure 5a). Fruit shape, the ratio of height to width, had a positive effect on percent nutlet fusion; a one standard deviation increase in shape (fruit shape becomes more elongate) was associated with a 0.545 unit increase in the expected log odds of percent nutlet fusion (se = 0.067, Profile CI = 0.408–0.681), when fruit volume was held at an average value, corresponding to a predicted probability of 63.3% (Table 5, Figure 5b).

**TABLE 5:** % nutlet fusion within fruit model summary.

	Fixed effects	Estimate	Std. error	Lower confidence interval	Upper confidence interval
	(intercept)	0.807	0.338	0.164	1.407
	Fruit volume	0.514	0.103	0.328	0.715
	Fruit shape	0.545	0.067	0.408	0.681

*Note*: A mixed‐effects generalized linear regression model was fit using a binomial error distribution and logit link function on 1726 observations. The model structure included the proportion of nutlet fusion per fruit (pfused/nutnum) as the dependent variable and fruit volume (frVS) and fruit shape (hwS) as fixed effects. Species identity was included as a random effect (*n* = 38, variance = 3.628, SD = 1.905). An observation‐level random effect (OLRE, Ucode) was included to account for overdispersion. Population was also included as a random effect, nested in species, to control for multiple populations of the same species, but not interpreted due to not all species being sampled from multiple populations. BIC = 5723, Pseudo‐R^2^ (fixed effects) = 0.08, Pseudo‐*R* ^2^ (total) = 0.52. Fixed‐effect estimates are in log‐odds. Bootstrap confidence intervals are given.

**FIGURE A6:**
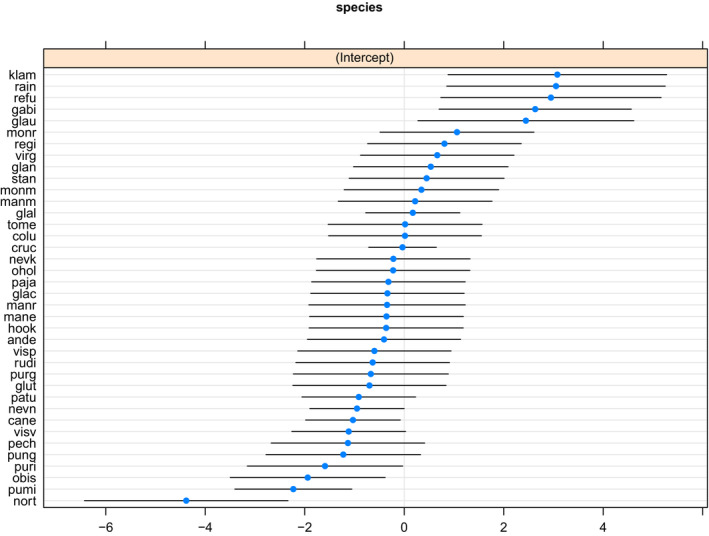
Nutlet fusion random effect. Species were specified as a random effect for the fruit fusion model. The plot shows individual and species‐level deviations from the intercept. Each line represents a different species.

The variation in proportion of nutlet fusion due to species was 3.63 (SD = 1.997, Table 4). The among‐species random‐effect standard deviation (in log‐odds of seed fusion) is comparable to the magnitude of the fixed effects and is greater than the largest single fixed effect, 0.545, the effect of shape. For species‐level deviations from the intercept, *A. nortensis* was the most negatively different, and *A. klamathensis* the most positively different (Figure A6).

### 3.3 Seed viability within fruits model

**FIGURE 6:**
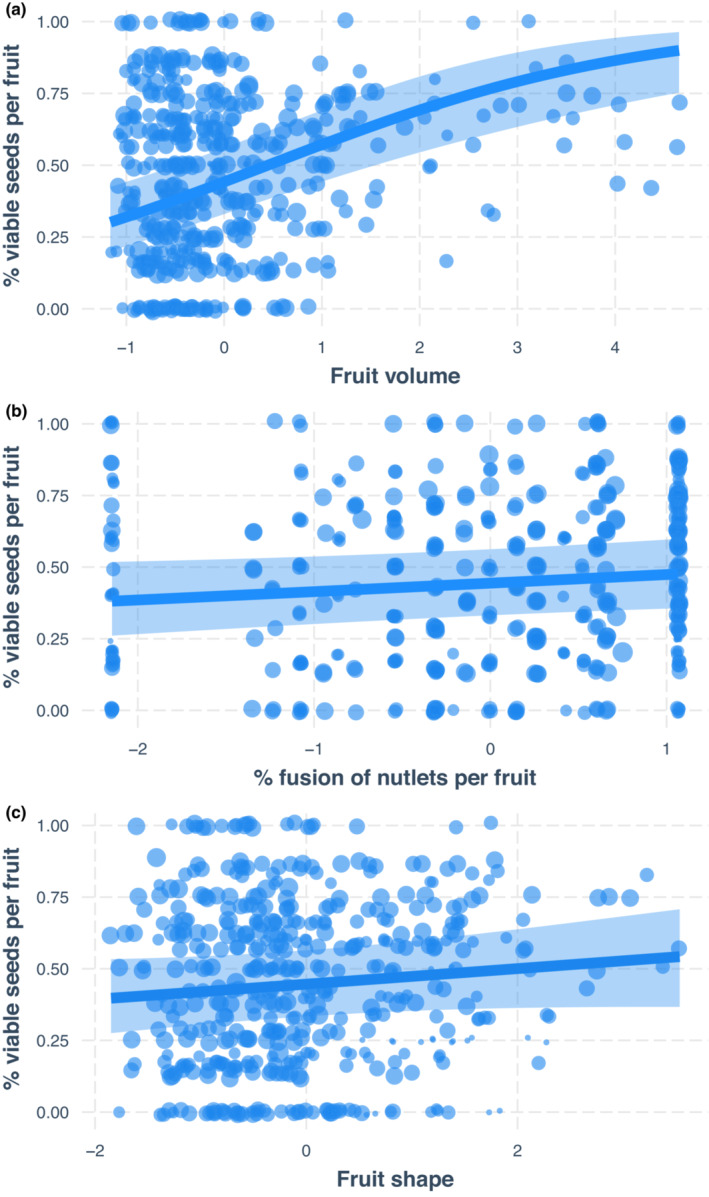
Regression estimate for the fruit viability model, with observations plotted. Predicted probability of percent viable seeds per fruit by fruit volume (in log‐odds). Light blue ribbon corresponds to a 95% confidence interval. (a) Predicted probability of percent viable seeds per fruit by fruit volume (in log odds). (b) Predicted probability of % viable seeds per fruit by % fused nutlets per fruit; c) Predicted probability of percent viable seeds per fruit by fruit shape ratio (in log‐odds). Low values of height/width represent oblate‐shaped fruit, low‐medium values represent depressed globose fruit, medium values represent globose fruit, and high values represent obloid/ellipsoid fruit.

The estimated effect (in log‐odds scale) of fruit volume on percent seed viability per fruit was 0.522 (se = 0.107, Profile CI: 0.314–0.736, Table 6, Figure 6a), or a predicted probability of 62.8%. Percent nutlet fusion and fruit shape may have little effect on percent seed viability because the confidence intervals include zero. The lower confidence intervals are very close to zero, so we mention and interpret the effects with caution. The estimated effect of nutlet fusion on percent seed viability was 0.121 and a predicted probability of 53% (se = 0.068, Profile CI: −0.012, 0.256, Table 6, Figure 6b). Fruit shape had a similar effect on percent seed viability as % nutlet fusion (est. = 0.109, se = 0.077, Profile CI: −0.042, 0.261, Table 6, Figure 6c).

**TABLE 6:** Viability model summary.

Fixed effects	Estimate	Std. error	Lower confidence interval	Upper confidence interval
(intercept)	−0.230	0.242	−0.741	0.277
Fruit volume	0.522	0.107	0.314	0.736
% fusion	0.121	0.068	−0.012	0.256
Fruit shape	0.109	0.077	−0.042	0.261

Note: A mixed‐effects generalized linear regression model was fit using a binomial error distribution and logit link function on 479 observations. The model structure included the proportion of viable seeds per fruit (full/nutnum) as the dependent variable and fruit volume (frVS), proportion of nutlet fusion per fruit (pfusedS), and fruit shape (frshS) as fixed effects. Species identity was included as a random effect (*n* = 15, variance = 0.8385, SD = 0.916). An observation‐level random effect (OLRE, Ucode) was also included. BIC = 1738.89, Pseudo‐*R* ^2^ (fixed effects) = 0.079, Pseudo‐*R* ^2^ (total) = 0.249. Fixed‐effect estimates are in log‐odds. Profile confidence intervals are given.

**FIGURE A7:**
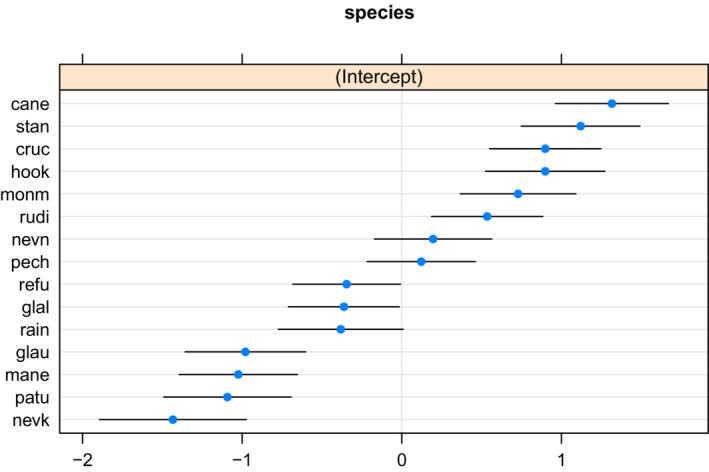
Seed viability random effect. Species were specified as a random effect for the seed viability model. The plot shows species‐level deviations from the intercept. Each line represents a different species.

The among‐species variance was 0.839, and the standard deviation was 0.916, which is higher in magnitude to the effect of fruit volume, 0.522. The random‐effects plots show the group‐level deviations from the fixed intercept, which illustrates how much a particular species intercept is shifted toward or away from the fixed intercept (Figure A7). For species‐level deviations from the intercept, *A. nevadensis* subsp. *knightii* (nevk) was the most different, as were *A. patula* (patu), *A. canescens* (cane), and *A. stanfordiana* (stan).

## 4 DISCUSSION

Our motivation for this study was determining whether *Arctostaphylos* fruit and seed traits vary in a way that could influence scatter‐hoarding rodent decisions regarding long‐term persistent seed banks. Almost all studies conducted on the survival of seeds in caches have contrasted seeds with no dormancy against short‐term (<1 year) dormancy (Chauvet et al., 2004; Fox, 1982; Hadj‐Chikh et al., 1996; Jansen et al., 2006; Xiao et al., 2009) in contrast to the long‐term, fire‐response seed dormancy in *Arctostaphylos*. We found that *Arctostaphylos* species increase their likelihood of long‐term survival by manipulating the perception of size and food value in their fruit in three ways. Contrasts in fruit shape reflect different fruit trends and plants have varied both fusion of nutlets and viability of seed in the context of both size and shape. Traits modifying the balance between consumption and caching by rodents include seed dormancy, size, and other aspects of food value (Chang et al., 2009; Sundaram et al., 2017; Kuprewicz & García‐Robledo, 2019; Vander Wall et al., 2019), as well as to risks imposed by handling time, predators, or other costs (Lichti et al., 2017). But scatter‐hoarding rodents are proficient at finding seeds in the soil (Vander Wall, 1995) and their proficiency acts on plants to adjust fruit and seed traits to both attract rodents and bury their seed, but also to limit the rates of their consumption (Jacobs, 1992; Vander Wall, 2010, 2019; Zwolak & Crone, 2012; Lichti et al., 2017; Sundaram et al., 2017; Vander Wall et al., 2019).

The probabilities of the long‐term survival of cached seed should be limited given the size of the seed, even though traits of fruit and seed would be selected to manipulate the behavior of seed predators to permit the sustainability of seed banks (Hulme, 1998; Lichti et al., 2017). Size is one of the most important characteristics of seed value, and fruit in *Arctostaphylos* ranges among species from a few mm to a little over 16 mm in diameter, with seed size paralleling fruit size. While seed size increases with fruit size, so do endocarp thickness and fusion of nutlets together. Rodents cache whole fruit of *Arctostaphylos* (Parker, 2015), although they will cache seed (nutlets) found in coyote or bear scat as well (Moore & Vander Wall, 2015), and once in the soil, the fruit outer pericarp breaks down and nutlets that are not fused together become much smaller food resources for rodents. The separation of nutlets represents a hidden trait in *Arctostaphylos* that rodents discover upon recovering caches. These now independent resources vary in degrees of nutlet fusion, and thus size. Variation in the fusion of nutlets together within fruit represents a trait that may increase the probability of seed survival in the soil by some seed escaping notice of rodents when retrieving caches. If we compare species of this genus to other relatives in the Arbutoideae, the Ericaceae subfamily to which they belong, *Arctostaphylos* appears to be a more recently derived taxon within the subfamily (Hileman et al., 2001) and close relatives, like *Comarostaphylis*, *Ornithostaphylos*, and *Xylococcus*, have small nutlets that are all fused into single globose structures (Parker & Stickrod, 2022). This suggests that the loss of nutlet fusion is a derived character in *Arctostaphylos*, the only Arbutoid genus with persistent seed banks. Nutlet fusion is also positively related to fruit size.

Fruits of *Arctostaphylos* also are characterized by frequent empty seed chambers. The number of viable seeds per fruit ranged from zero to all seeds being viable and that variation occurred within individuals, populations, and species. While percent viability ranges among species, all taxa demonstrate large variability in the number of viable seed per fruit. Empty seed chambers have been interpreted as a way plants reduce seed predation (Fuentes & Schupp, 1998; Perea et al., 2013). While rodents in some cases may learn to distinguish among individual filled and empty nutlets (García et al., 2000; Muñoz & Bonal, 2008b), combining this trait with variable nutlet fusion nonetheless may reduce that learning curve and provide escape for some seed and permit maintenance of persistent seed banks.

An interesting finding in surveying variation in *Arctostaphylos* fruit is that shape appears to influence the degree of nutlet fusion and to a lesser extent, seed viability. Percent seed viability per fruit may be slightly higher for obloid/ellipsoid, roughly globose‐shaped fruit, while viability declines for the oblate or depressed‐globose‐shaped fruit. Globose fruits tend to have high proportions of nutlet fusion, where all seeds are coalesced into a single structure, and may provide consistent perceived reward sizes for scatter‐hoarding rodents and therefore increase their perceived value. Because scatter‐hoarding rodents may preferentially disperse larger fruit, the plants may have been selected to invest more resources in producing viable seeds per fruit, as the high level of fusion found in large fruit ensures that the entire fruit is dispersed (Fuentes & Schupp, 1998, Perea et al., 2013). These larger fruits have thicker endocarps overall that impose higher handling costs, further reinforcing burial over immediate consumption (Vander Wall, 2010). Even when the exocarp disintegrates upon burial of the seeds, the fused nutlets conveniently hold all nutlets together in a single unit. While we could find no explicit experimental studies of different fruit shapes on rodent preferences, this is a direction for further study. The obloid/ellipsoid fruit may simply appear larger than their actual volume or may be easier to manipulate.

We expected environmental and life history variables to have an effect on the proportion of fused nutlets and viability per fruit, but they were not present in the best models (Table 2, Table 3) and correlation plots do not suggest strong patterns between these independent and dependent variables (Figures A2, A3, A4, A5). Previous studies demonstrated that temperature, elevation, precipitation, and latitude can affect fruit and seed mass, but these variables in our study do not drive differences in the degree of nutlet fusion and seed viability (Baker, 1972; Moles et al., 2007; Simpson et al., 2017). We also anticipated a relationship between proportion of fused nutlets and seed viability, but our data indicate that there is no clear pattern among all the species as a group.

Fruit size and shape were principal correlates on fruit nutlet fusion and viable seed per fruit in *Arctostaphylos*. Size usually sends a predictable signal to rodents about the amount of potential reward, resulting in preferential manipulation and dispersal for larger sized fruit. This is supported in studies of oak acorns (Muñoz & Bonal, 2008a; Zhang & Zhang, 2008; Sunyer et al., 2014), palms and tropical trees (Forget et al., 1998; Brewer, 2001; Jansen et al., 2002, 2004), pines (Wang & Chen, 2009), and mixed forests (Xiao et al., 2006). Larger fruit size comes with increased endocarp or other tissue thickness and may also be a constraint for some animals. In *Leucadendron* shrubs, rodent behavior was influenced by seed size and hull thickness; medium‐sized seeds with medium‐thick hulls were buried and small seeds with thin hulls were consumed in situ, while large seeds were not dispersed, likely a result of being too big and thick for the resident rodents to carry (Rusch et al., 2013). Seed size plays a distinct role in the context of scatter hoarding, in that particular rodent species will have different size preferences (Wang & Chen, 2009; Wang & Yang, 2020). Rodent preference for seed size or endocarp thickness may control the extent of burial versus immediate consumption (Dylewski et al., 2020; Rusch et al., 2013; Wang et al., 2012; Zhang & Zhang, 2008; Zhang et al., 2016). Our results are consistent with these studies in the sense of manipulating rodent behavior.

An additional issue is that, in *Arctostaphylos,* other traits also varied with fruit size and shape, specifically percent seed viability and nutlet fusion. Larger fruit tended to have higher percent viability while smaller fruit had lower nutlet fusion, but there was a lack of consistency in these trends for individual fruit. A consequence of the lack of consistency makes size alone a more ambiguous signal and underlies the patterns we observe and perhaps the success of this genus. Size and empty seed chambers have already been found as influences on scatter‐hoarding rodents (Fuentes & Schupp, 1998; Lichti et al., 2017). The difference in *Arctostaphylos* is that variations in both nutlet fusion and empty seed chambers are initially hidden from rodents when caching fruit. Combined, these traits may impact the economic decisions of rodents and permit greater seed escape. The density of seed banks in these species is negatively related to fruit and seed size (Parker & Ingalls, 2022), a reverse of trends found in the literature about size and caching (Thompson et al., 1993, 2001), suggesting that variation in size, nutlet fusion, and percent viable seed may play a critical role in seed escape and survival for these plants with long‐term seed dormancy and persistent soil seed banks.

## AUTHOR CONTRIBUTIONS

**Rebecca Emily Crowe:** Conceptualization (equal); data curation (lead); formal analysis (lead); investigation (lead); methodology (equal); visualization (equal); writing – original draft (lead); writing – review and editing (supporting). **V. Thomas Parker:** Conceptualization (equal); data curation (supporting); formal analysis (supporting); investigation (supporting); methodology (equal); visualization (equal); writing – original draft (supporting); writing – review and editing (lead).

## FUNDING INFORMATION

Yerba Buena CNPS, Cal Bot Soc, NCB, and Orange County CNPS provided funding for travel to conferences to share this research. VTP received funding from the SF State Office of Research and Sponsored Projects for this work.

## CONFLICT OF INTEREST

The authors declare that there are no competing interests.

## DATA AVAIALABILITY STATEMENT

Morphological data and viability data are available in the Mendeley Data repository, DOI: https://doi.org/10.17632/28c4zjb36h.1, (Crowe, 2023). Voucher specimens are curated at the Harry D. Thiers Herbarium (SFSU) with duplicates at the University of California, Irvine Herbarium (IRVC).

## APPENDIX A

**TABLE A1:** Number of populations, individual plants, and fruit sampled per *Arctostaphylos* species.

Species	Population codes	# Of populations	# Of plants (listed in order, by pop.)	# Of fruit (listed in order, by pop.)
*A. andersonii*	andea	1	12	33
*A. canescens*	canea, caneb, canec	3	bulk, 10, 2	30, 33, 33
*A. columbiana*	colua	1	3	33
*A. crustacea* subsp. *crustacea*	cruca ‐ crucf	6	9, 8, bulk, bulk, bulk, bulk	33, 33, 33, 33, 33, 33
*A. gabilanensis*	gabia	1	bulk	33
*A. glandulosa* subsp. *cushingiana*	glaca	1	2	33
*A. glandulosa* subsp. *leucophylla*	glala, glalb, glalc	3	8, 3, 9	33, 33, 33
*A. glandulosa* subsp. *glandulosa*	glana	1	bulk	33
*A. glauca*	glaua, glaub	2	bulk, 10	33, 33
*A. glutinosa*	gluta	1	10	33
*A. hookeri*	hooka	4	4	30
*A. klamathensis*	klama	1	5	30
*A. manzanita* subsp. *elegans*	manea	1	11	33
*A. manzanita* subsp. *manzanita*	manma	1	4	33
*A. manzanita* subsp. *roofii*	manra	1	3	25
*A. montana* subsp. *montana*	monm	1	10	30
*A. montaraensis*	monr	1	7	32
*A. nevadensis* subsp. *knightii*	nevk	1	2	33
*A. nevadensis* subsp. *nevadensis*	nevna, nevnc, nevnd	3	bulk, 2, 10	33, 33
*A. nortensis*	norta	1	7	33
*A. obispoensis*	obisa	1	bulk	33
*A. ohloneana*	ohola	1	4	33
*A. pajaroensis*	pajaa	1	7	33
*A. patula*	patua, patub	2	8, bulk	40, 33
*A. pechoensis*	pecha	1	11	33
*A. pumila*	pumib, pumic	1	bulk, bulk	33, 33
*A. pungens*	punga	1	bulk	30
*A. purissima* subsp. *globosa*	purga	1	12	32
*A. purissima* subsp. *purissima*	puria	1	12	33
*A. rainbowensis*	raina	1	3	34
*A. refugioensis*	refua	1	12	33
*A. regismontana*	regia	1	4	33
*A. rudis*	rudia	1	bulk	33
*A. stanfordiana*	stana	1	bulk	30
*A. tomentosa*	tomeb	1	bulk	33
*A. virgata*	virga	1	5	33
*A. viscida subsp. pulchella*	vispa	1	11	33
*A. viscida subsp. viscida*	visvb, visvc	2	11, 10	33, 33

*Note*: Bulk collections refer to collection made from multiple individuals in a single population where all fruit were pooled and individual plants were not tracked. When # of fruit is <33, a fruit was lost over the course of being measured and was not able to be replaced.

**TABLE A2:** Environmental variables by species and overall.

Species	Elevation (m)	Precipitation (mm)	T_max_ (C)	Climatic water deficit	Latitude (decimal degrees)	Longitude (decimal degrees)
*A. andersonii*	584	1040	20	839	37.20	−122.20
*A. canescens*	764 (185)	891 (8)	20 (1)	684 (40)	39.36 (0.69)	−122.50 (0.21)
*A. columbiana*	1	980	14	596	39.49	−123.80
*A. crustacea* subsp. *crustacea*	529 (171)	791 (196)	21 (1)	816 (113)	36.95 (0.94)	−121.70 (0.56)
*A. gabilanensis*	692	505	21	935	36.71	−121.50
*A. glandulosa* subsp. *cushingiana*	1451	657	20	918	33.31	−116.90
*A. glandulosa* subsp. *leucophylla*	1471 (133)	658 (19)	20 (1)	843	33.33 (1.63)	−116.90 (1.21)
*A. glandulosa* subsp. *glandulosa*	597	719	24	925	36.11	−121.20
*A. glauca*	630 (31)	695 (23)	22 (2)	903	36.67 (0.53)	−121.40 (0.14)
*A. glutinosa*	518	1194	20	841	37.13	−122.20
*A. hookeri*	55	453	20	841	36.80	−121.70
*A. klamathensis*	1939	1840	12	336	41.20	−122.50
*A. manzanita* subsp. *elegans*	1030 (224)	916 (27)	19 (2)	661	39.82 (0.01)	−122.70 (0.01)
*A. manzanita* subsp. *manzanita*	1014 (216)	914 (26)	19 (1)	713	39.82 (0.01)	−122.70 (0.01)
*A. manzanita* subsp. *roofii*	935	1332	20	425	41.15	−122.70
*A. montana* subsp. *montana*	200	1225	21	765	37.97	−122.60
*A. montaraensis*	374	890	18	715	37.57	−122.50
*A. nevadensis* subsp. *knightii*	603 (20)	2675 (12)	17 (1)	365	41.90 (0.02)	−124.00 (0.02)
*A. nevadensis* subsp. *nevadensis*	1567 (700)	2152 (389)	14 (1)	418	40.42 (1.04)	−121.80 (1.61)
*A. nortensis*	544 (65)	2617 (121)	16	345	41.88 (0.01)	−124.10 (0.01)
*A. obispoensis*	664	887	21	946	35.36	−120.70
*A. ohloneana*	518	1194	20	842	37.12	−122.20
*A. pajaroensis*	55	453	20	841	36.80	−121.70
*A. patula*	1380 (333)	1092 (399)	18 (2)	582	37.63 (3.95)	−120.00 (2.92)
*A. pechoensis*	239	620	23	948	35.24	−120.80
*A. pumila*	33	377	18	841	36.68	−121.80
*A. pungens*	1150	492	20	931	36.40	−120.70
*A. purissima* subsp. *globosa*	253	597	21	1022	34.51	−120.30
*A. purissima* subsp. *purissima*	143	406	21	983	34.70	−120.40
*A. rainbowensis*	412 (47)	430 (2)	24	1059	33.44 (0.01)	−117.20 (0.01)
*A. refugioensis*	702	808	23	959	34.54	−120.10
*A. regismontana*	530	998	22	729	37.43	−122.30
*A. rudis*	143	406	21	983	34.70	−120.40
*A. stanfordiana*	491	879	21	626	38.34	−122.20
*A. tomentosa*	33	377	18	841	36.68	−121.80
*A. virgata*	345	1080	19	786	37.97	−122.70
*A. viscida subsp. pulchella*	803 (72)	846 (29)	20 (1)	790	39.82	−122.70 (0.01)
*A. viscida subsp. viscida*	1116.00 (96.74)	1495	19 (1)	523	40.22 (1.03)	−121.70 (0.92)
**Overall mean (SD)**	**717.00 (527.10)**	**973**	**20 (3)**	**755**	**37.42 (2.5)**	−*121.40 (1.88)*

*Note*: Means (standard deviation) are given when more than one population exists.

**TABLE A3:** Summary of morphological traits.

*Arctostaphylos* Species	Nutlet Fusion Mean (SD)	Height (mm) Mean (SD))	Width (mm) Mean (SD)	Shape Mean (SD)	#Nutlets Mean (SD)	Fruit Volume Mean (SD)
*A. andersonii*	0.50 (0.25)	5.54 (0.85)	7.49 (1.18)	0.74 (0.08)	7.06 (0.86)	172.5 (73.82)
*A. canescens*	0.33 (0.31)	4.88 (1.07)	7.43 (1.59)	0.66 (0.1)	7.84 (0.94)	157 (90.64)
*A. columbiana*	0.54 (0.23)	5.55 (0.47)	8.89 (0.81)	0.63 (0.04)	8.15 (0.83)	233.5 (60.15)
*A. crustacea* subsp. *crustacea*	0.61 (0.24)	6.02 (0.83)	8.22 (0.98)	0.74 (0.1)	6.5 (0.89)	219.5 (72.56)
*A. gabilanensis*	1.00 (0.02)	9.11 (1.31)	9.99 (1.05)	0.91 (0.08)	6.61 (0.9)	491.7 (159.1)
*A. glandulosa* subsp. *cushingiana*	0.64 (0.27)	7.25 (0.87)	8.79 (0.93)	0.83 (0.1)	7.09 (0.72)	301.1 (86.43)
*A. glandulosa* subsp. *leucophylla*	0.67 (0.26)	6.31 (1.16)	8.74 (1.53)	0.73 (0.07)	6.97 (0.85)	273.6 (138.6)
*A. glandulosa* subsp. *glandulosa*	0.80 (0.21)	7.03 (0.95)	8.90 (1.03)	0.79 (0.09)	7.03 (0.95)	300.4 (98.02)
*A. glauca*	1.00 (0.00)	13.41 (1.25)	12.88 (1.41)	1.05 (0.08)	6.33 (0.84)	1195 (335.8)
*A. glutinosa*	0.44 (0.29)	6.45 (0.91)	9.51 (0.96)	0.68 (0.09)	7.55 (0.9)	312 (92.98)
*A. hookeri*	0.49 (0.31)	4.68 (0.44)	6.13 (0.49)	0.76 (0.03)	6 (1.08)	93.71 (20.92)
*A. klamathensis*	1.00 (0.00)	5.04 (0.96)	4.88 (0.74)	1.03 (0.1)	4.47 (0.9)	67.23 (30.11)
*A. manzanita* subsp. *elegans*	0.71 (0.22)	8.54 (1.41)	11.39 (1.62)	0.75 (0.07)	6.64 (0.55)	612.5 (252)
*A. manzanita* subsp. *manzanita*	0.73 (0.25)	7.52 (1.07)	10.80 (1.17)	0.7 (0.08)	7.46 (1.2)	473.8 (161.9)
*A. manzanita* subsp. *roofii*	0.70 (0.27)	7.65 (1.18)	8.53 (0.90)	0.9 (0.13)	5.16 (0.55)	298.2 (93.32)
*A. montana* subsp. *montana*	0.73 (0.22)	6.65 (0.98)	8.84 (1.13)	0.76 (0.11)	6.87 (0.94)	280.6 (95.37)
*A. montaraensis*	0.77 (0.22)	5.05 (0.59)	7.79 (0.88)	0.65 (0.05)	8.06 (0.98)	165.4 (52.68)
*A. nevadensis* subsp. *knightii*	0.56 (0.32)	5.91 (0.65)	7.95 (0.79)	0.75 (0.08)	6.67 (0.92)	199.7 (55.74)
*A. nevadensis* subsp. *nevadensis*	0.46 (0.35)	6.48 (0.74)	8.16 (0.78)	0.8 (0.1)	6.05 (1.06)	229.5 (57.54)
*A. nortensis*	0.00 (0.00)	4.47 (0.64)	7.57 (0.80)	0.59 (0.05)	8.18 (1.01)	138.8 (49.18)
*A. obispoensis*	0.15 (0.19)	6.42 (0.66)	10.67 (1.19)	0.6 (0.04)	6.97 (0.73)	394.2 (115.6)
*A. ohloneana*	0.48 (0.31)	4.92 (0.87)	7.31 (1.19)	0.68 (0.08)	6.15 (0.97)	146.9 (65.16)
*A. pajaroensis*	0.42 (0.30)	4.51 (0.44)	6.95 (0.80)	0.65 (0.08)	6.09 (0.95)	116.6 (33.95)
*A. patula*	0.48 (0.36)	6.45 (1.82)	8.18 (1.70)	0.78 (0.09)	5.8 (1.03)	259.3 (151)
*A. pechoensis*	0.39 (0.34)	7.03 (1.11)	10.32 (1.62)	0.68 (0.07)	7.42 (0.79)	416.4 (182.2)
*A. pumila*	0.17 (0.24)	5.48 (0.58)	7.53 (0.81)	0.73 (0.07)	5.41 (0.78)	166.7 (49.71)
*A. pungens*	0.45 (0.42)	7.99 (1.00)	9.72 (1.24)	0.82 (0.04)	5.77 (0.86)	411.5 (136.8)
*A. purissima* subsp. *globosa*	0.54 (0.41)	5.37 (0.80)	5.90 (0.85)	0.93 (0.21)	4.72 (0.77)	100.2 (36.53)
*A. purissima* subsp. *purissima*	0.19 (0.28)	4.80 (0.71)	6.73 (0.73)	0.71 (0.08)	5.61 (0.93)	117.2 (39.15)
*A. rainbowensis*	1.00 (0.00)	7.72 (0.80)	7.99 (0.61)	0.97 (0.06)	4.44 (0.56)	262.4 (65)
*A. refugioensis*	1.00 (0.00)	10.22 (1.45)	10.63 (0.97)	0.97 (0.15)	7.18 (0.77)	611.3 (139.2)
*A. regismontana*	0.70 (0.27)	4.52 (0.60)	6.85 (0.79)	0.66 (0.05)	7.24 (0.83)	115.1 (39.78)
*A. rudis*	0.49 (0.35)	7.02 (1.12)	9.83 (0.84)	0.71 (0.09)	6.61 (1.32)	363.7 (113.5)
*A. stanfordiana*	0.80 (0.19)	7.34 (0.69)	9.32 (0.81)	0.79 (0.11)	7.1 (0.66)	335.3 (60.96)
*A. tomentosa*	0.70 (0.26)	6.80 (0.81)	8.48 (1.11)	0.81 (0.1)	6.15 (0.67)	263.7 (82.25)
*A. virgata*	0.68 (0.26)	5.37 (0.73)	8.68 (0.87)	0.62 (0.07)	7.18 (1.04)	217.5 (68.85)
*A. viscida subsp. pulchella*	0.50 (0.32)	6.68 (0.87)	8.99 (0.77)	0.74 (0.08)	6.67 (0.85)	287.8 (78.17)
*A. viscida subsp. viscida*	0.30 (0.32)	5.28 (0.71)	7.88 (0.87)	0.67 (0.08)	7 (0.99)	176.42 (54.2)
**Overall Means (SD)**	*0.57 (0.35)*	*6.47 (2.03)*	*8.51 (1.84)*	0.76 (0.14)	6.59 (1.24)	285.7 (240.8)

*Note*: Nutlet fusion = proportion of nutlets fused together. Height and width refer to fruit measurements; Shape = Width/height; # Nutlets = mean # nutlets found per fruit; Fruit volume calculated from height and width. Sample size *N* = (30‐)33 for each species.
